# The origin of hyperferroelectricity in Li*B*O_3_ (*B* = V, Nb, Ta, Os)

**DOI:** 10.1038/srep34085

**Published:** 2016-10-03

**Authors:** Pengfei Li, Xinguo Ren, Guang-Can Guo, Lixin He

**Affiliations:** 1 Key Laboratory of Quantum Information, University of Science and Technology of China, Hefei, 230026, China; 2 Synergetic Innovation Center of Quantum Information and Quantum Physics, University of Science and Technology of China, Hefei, 230026, China

## Abstract

The electronic and structural properties of Li*B*O_3_ (*B* = V, Nb, Ta, Os) are investigated via first-principles methods. We show that Li*B*O_3_ belong to the recently proposed hyperferroelectrics (hyperFEs), i.e., they all have unstable longitudinal optic phonon modes. Especially, the ferroelectric-like instability in the metal LiOsO_3_, whose optical dielectric constant goes to infinity, is a limiting case of hyperFEs. Via an effective Hamiltonian, we further show that, in contrast to normal proper ferroelectricity, in which the ferroelectric instability usually comes from long-range coulomb interactions, the hyperFE instability is due to the structure instability driven by short-range interactions. This could happen in systems with large ion size mismatches, which therefore provides a useful guidance in searching for novel hyperFEs.

The switchable polarization of ferroelectrics (FEs) made them an important class of materials for modern device applications. However, in traditional proper FEs, the electric polarization is very sensitive to the domain wall structures and electric boundary conditions^1^. This is even more severe in the case of ferroelectric thin films^2^,^3^, where the depolarization field may easily destroy the polarization states. Recently in a seminal work^4^, Garrity, Rabe and Vanderbilt (GRV) showed that a class of recently discovered hexagonal *ABC* semiconducting FEs^5^ have very robust polarization properties even when the depolarization field is unscreened. For example, they can remain polarized down to single atomic layers when interfaced with normal insulators, and are therefore given the name of *hyperferroelectrics* (hyperFEs). These properties are extremely important for designing modern devices which utilize the FE thin films^6^,^7^. GRV further showed that the extraordinary behavior of hyperFEs is because they have an unstable longitudinal optic (LO) mode besides the transverse optic (TO) mode instability.

It has been proposed that in the hexagonal *ABC* hyperFEs, the imaginary LO phonon frequency is due to the small LO-TO splitting, which further arises from their small energy gaps – thus large optical dielectric constants *ε*_∞_ – as well as small Born effective charges^4^. However, in this Letter, we show that LiNbO_3_, LiTaO_3_ are also hyperFEs, because they have unstable LO phonon modes as well, despite that they have relative small *ε*_∞_ and large mode effective charges, in contrast with the above-mentioned *ABC* hyperFEs. This poses an interesting question: is there a more general (fundamental) driving mechanism for hyperFEs, besides the small LO-TO splitting scenario disclosed by GRV for hexagonal *ABC* FEs? We will answer the question in this work using the Li*B*O_3_-type materials.

LiNbO_3_ and LiTaO are two important FEs which have been investigated intensively in the past years^8^,^9^,^10^,^11^,^12^,^13^. The FE transition of these materials is believed to be of order-disorder character. Shown in Fig. 1 is the hypothetical paraelectric (PE) structure of Li*B*O_3_ (*B* = Nb, Ta), resulting from an average of the disordered structure above *T*_*c*_. The paraelectric (PE) structure belongs to the 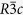 space group, whereas the FE structure is rhombohedral, and belongs to the space group *R*3*c*. In the FE phase, the Li, O, and *B* ions distort from their central symmetric positions, which induces an electric polarization along the trigonal axis. Inbar and Cohen studied the electronic and structural properties of LiNbO_3_ and LiTaO_3_^13^. They found large hybridization between the transition-metal *B* atoms and the oxygen 2*p* states, similar to perovskite FEs. It has thus been suggested that the ferroelectricity in LiNbO_3_ and LiTaO_3_ is due to long-range Coulomb interactions. Interestingly, very recently it has been found that LiOsO_3_, even though being a metal, also has FE-like structural transitions^14^. It is very puzzling where the FE-like structure of LiOsO_3_ comes from, since the long-range Coulomb long-range interactions should be screened in the metallic states. It has been argued that the lattice distortion is due to ionic size mismatch at the Li site^15^,^16^,^17^. On the other hand, Liu *et al.* argued that the distortion is due to the lack of electric screening along the polar direction^18^.

In this work, we investigate the FE properties of Li*B*O_3_-type compounds, where *B* = V, Nb, Ta and Os using first-principles methods (see Methods). We show that Li*B*O_3_ are hyperFEs, and there are two co-existing and yet distinct FE mechanisms in Li*B*O_3_, namely the long-range Coulomb interactions due to *B* ions, and short-range structural instability due to Li ions. Especially we show that the instability of Li ions is responsible for the hyperFE behavior of Li*B*O_3_. The FE-like structural transition in metallic LiOsO_3_^14^ is nothing special, but has the same mechanism of other Li*B*O_3_ compounds. In this sense, LiOsO_3_ can be viewed as a special hyperFEs in the limit of *ε*_∞_ → ∞. Via an effective Hamiltonian model, we further clarify that the microscopic origin of hyperFEs is from the instability driven by short-range interactions. These results provide a strong guidance in searching for novel hyperFEs.

## Results

The FE phase transitions can be understood by the lattice dynamics of their high-symmetry phase. For FEs, the high-symmetry phase has at least one unstable TO mode. The frequencies of TO can be calculated using first-principles methods in bulk materials in the absence of macroscopic electric field 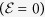
4. If the depolarization field is unscreened, corresponding to the case of electric displacement *D* = 0, the structure instability is determined by the LO modes, which can be obtained by adding to the dynamic matrix a non-analytic long-range Coulomb term (known as the LO-TO splitting)^1^,^19^ that schematically takes the form 4πZ*^2^/Ω*ε*_∞_, where Z* is the Born effective charge and Ω is the volume of the unit cell. In normal FEs, such as PbTiO_3_, BaTiO_3_, etc., due to their large Born effective charges and relatively small *ε*_∞_, the LO-TO splittings are huge, such that all LO modes are stable^1^. Therefore they lose ferroelectricity if the depolarization field is not well screened. In contrast, in the *ABC* hexagonal hyperFEs (e.g., LiZnAs) as discussed by GRV^4^, the LO-TO splittings are small, such that even the LO modes can become unstable. Consequently, the polarization in these materials is very robust against the depolarization field.

The FE materials with small LO-TO splittings are the most obvious candidates for hyperFEs. Therefore, it is a natural attempt to look for the hyperFEs in materials with (i) small band gap, or equivalently large electronic dielectric constant *ε*_∞_; (ii) small mode effective charges. Indeed, the hyperFEs found by GRV all satisfy these conditions^4^. However, as demonstrated below, LiNbO_3_ and LiTaO_3_ are also hyperFEs, i.e., having unstable LO phonon modes, despite that they have large band gaps, relatively small optical dielectric constants, and large mode effective charges.

The calculated band gaps (via DFT-LDA) of Li*B*O_3_ are listed in Table 1. We see that in the PE phase, the LDA calculated band gaps of LiNbO_3_ and LiTaO_3_ are 2.2 eV and 3.0 eV respectively, and increase to 2.9 eV and 3.6 eV in the FE phase. These, as usual, are underestimated compared to the experimental values of 3.78 eV for LiNbO_3_^20^ and 4.7 eV for LiTaO_3_^21^, measured in the FE phase. Also, one may note that the band gaps of these two materials are comparable to those of perovskite FEs, but much larger than the band gaps of *ABC* hexagonal hyperFEs, which are around 0.5–1 eV^4^. LiVO_3_ has a relatively small LDA band gap, which is only about 0.4 eV in the PE phase, but increase significantly to 1.9 eV in the FE phase. LiOsO_3_ is a metal^14^. The calculated optical dielectric constant 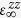 along the (polar) *z* axis and Born effective charges are also listed in Table 1. The calculated 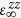 of LiNbO_3_ is 7.3 in the PE phase, and 5.8 in the FE phase, compared to the experimental value 4.6 measured in the FE phase^22^. The calculated 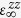 of LiTaO_3_ are 5.8 in the PE phase and 5.3 in the FE phase. Both materials have similar 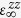 to those of perovskite FEs, e.g., PbTiO_3_ and BaTiO_3_. The dielectric constant of LiVO_3_ is somehow larger, approximately 19 in the PE phase, but drops to approximately 6.4 in the FE phase, consistent with the corresponding band gaps in both phases. LiOsO_3_ is a metal, and therefore its 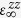 diverges. Finally we discuss the calculated atomic Born effective charges for LiVO_3_, LiNbO_3_ and LiTaO_3_. The effective charge of Li ions is approximately 1.0, suggesting that Li is totally ionized. The effective charges of V, Nb, and Ta ions are approximately 13, 9, and 8 respectively, which are anomalously large compared to their valence electron charges, but similar to those of perovskite FEs. It is usually believed that the anomalous effective charges introduce large long-range Coulomb interactions, which further lead to spontaneous electric polarizations in FEs^13^,^23^.

To study the structural instabilities in Li*B*O_3_, we calculated the phonons of Li*B*O_3_ at Γ point in the PE phases using a 10-atom unit cell. We focus on the *A*_2*u*_ modes which are associated with the FE structural transitions. For these modes, the phonon frequencies for both the TO mode and LO modes are calculated. The results are summarized in Table 2. As a comparison, we also present the results for some normal perovskite FEs, including PbTiO_3_, BaTiO_3_, NaNbO_3_, and KNbO_3_. We note that the results for the perovskites are all obtained at their cubic structures, and therefore these results should not be compared to the experimental values directly. As listed in Table 2, LiVO_3_, LiNbO_3_, LiTaO_3_, and LiOsO_3_ all have very strong instable TO modes. Especially the phonon frequencies of LiNbO_3_ are very close to those calculated in refs 24 and 25. This is consistent with the proposal that LiTaO_3_ and LiNbO_3_ are order-disorder FEs^8^,^9^,^10^,^11^,^12^, in which the centrosymmetric structures have much higher energies than the distorted structures. The calculated mode effective charges for LiVO_3_, LiNbO_3_, and LiTaO_3_ are approximately 20, 9, and 6, respectively. The Born effective charges are ill-defined for LiOsO_3_, which is a metal. We also present the electric polarizations for their FE phase. The calculated electric polarization of LiNbO_3_ and LiTaO_3_ are 1.00 C/m^2^ and 0.72 C/m^2^ respectively, which are somehow larger than the experimental values 0.77^26^ and 0.50 C/m^2 ^27 correspondingly. These values are comparable to those of perovskite FEs.

**Figure 2:**
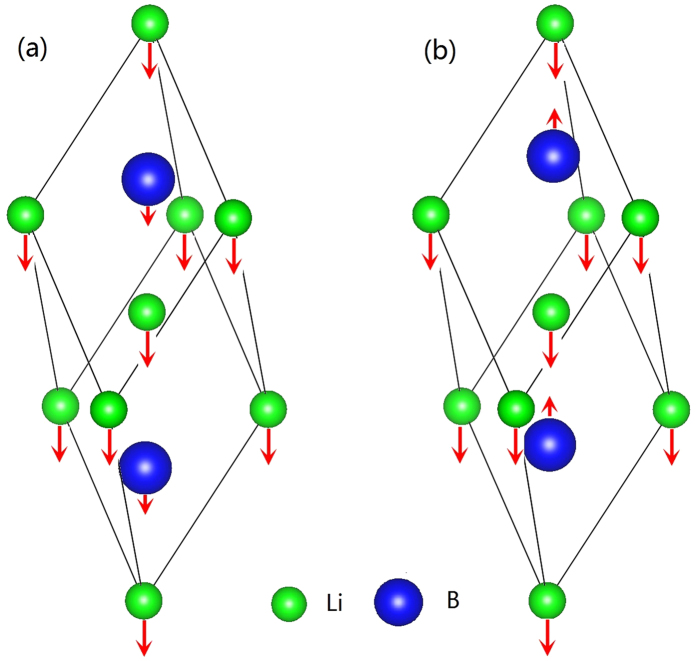
The schematic phonon patterns showing the atomic displacements of Li and *B* atoms for the soft (**a**) TO mode, and (**b**) LO mode. For clarity, we neglect the oxygen atoms.

The LO phonon frequencies are calculated by diagonalizing the resultant matrix obtained by adding the non-analytic terms to the dynamic matrix^1^,^19^ i.e.,


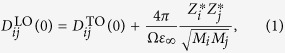


where 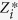, 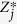 are the atomic effective charges, and *M*_*i*_, *M*_*j*_ are the atomic masses. The results are also given in Table 2. Remarkably, all calculated Li*B*O_3_ compounds have soft LO modes, indicating that they are hyperFEs, similar to the *ABC* hexagonal FEs but in contrast to the perovskite ones. We also calculate the phonon dispersion of high symmetry 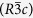 LiNbO_3_ and LiTaO_3_ for **q** along *X*-Γ-*Z* directions (see Supplementary Information), and the results are consistent with those calculated from Eq. 1 at Γ point. For LiOsO_3_, the TO modes and LO modes have the same frequencies, because its 1/*ε*_∞_ = 0. These results are quite surprising, given that the dielectric constants *ε*_∞_ of LiNbO_3_ and LiTaO_3_ are relative small, and the mode effective charges are quite large, similar to those of the traditional perovskite FEs, such as PbTiO_3_, BaTiO_3_ etc. One may expect that the LO-TO splitting 4πZ*^2^/Ω*ε*_∞_ would stabilize all LO modes. To understand the origin of the soft LO modes, we analyze the eigenvectors of the soft *A*_2*u*_ modes of Li*B*O_3_, for both TO modes and LO modes. The atomic displacements of the soft TO and LO phonons are shown in Fig. 2(a,b), respectively. In TO modes, Li ions and *B (B* = V, Nb, Ta) ions move in the same direction, whereas the O ions (not shown) move along the opposite direction. The Li ions have the largest displacement, where *B* and O ions also have significant contributions. For LO modes, the displacements of O ions along the *c* axis are somehow suppressed. Surprisingly, the displacements of *B* ions reverse from those of the TO mode, i.e., opposite to the polarization direction! These results show that the phonon eigenvectors are very sensitive to the electric boundary conditions, and are very different for TO modes and LO modes. Therefore, adding the simple correction term 4πZ*^2^/Ω*ε*_∞_ to the TO modes would significantly overestimate the LO-TO splitting, and falsely stabilizes all LO modes. One has to make the non-analytical corrections to the dynamic matrices themselves. As we see from Eq. 1 the corrections to the V, Nb, Ta ions are very large due to their large effective charges; whereas the corrections for Li ions are small, because Z*(Li) ≈ 1, is small. Therefore, the LO modes of Li*B*O_3_ can remain soft by altering their mode patterns.

From the above analysis, one can see there are two different contributions to the ferroelectricity in Li*B*O_3_. One is the long-range Coulomb interactions due to large Born effective charges arising from the *B* ions, which are very sensitive to the electric boundary conditions, just like normal proper FEs; the second is short-range instability due to the large size mismatch between the Li and *B* ions^15^, which is robust against the electric boundary conditions. Especially for metallic LiOsO_3_, the FE-like structural transition is induced by the short-range instability of Li alone, because the long-range Coulomb interactions is screened.

Additional evidence for the hyperferroelectricity of Li*B*O_3_ materials comes from direct calculations of the electric polarization under the boundary condition *D* = 0. To calculate this quantity, we expand the free energy of the system around the high symmetry structure^4^, as a function of the LO phonon mode and the electric field 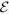, under the constrain 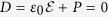,


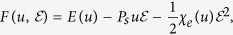


where *E*(*u*) is the total energy as function of *u* under 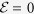, which can be directly calculated by the first-principles method. 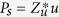 is the spontaneous polarization, where 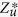 is the LO phonon mode Born effective charge. *χ*_*e*_(*u*) is the zero-field electron susceptibility as a function of *u*. Because the lattice distortion is rather small under *D* = 0, we take *χ*_*e*_(*u*) ≈ *χ*_*e*_(0). The minima of 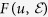 gives the structure displacement *u*_0_ under *D* = 0. The corresponding spontaneous polarization is then calculated as Z*u_0_. The obtained results are listed in Table 2. As expected, for normal FEs PbTiO_3_, BaTiO_3_, NaNbO_3_, and KNbO_3_, the spontaneous polarizations are all zero under *D* = 0. In contrast, for LiVO_3_, LiNbO_3_, and LiTaO_3_, the spontaneous polarizations under *D* = 0 are about one tenth of those under 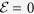, but still significant for applications. We also investigate an artificial LiTaO_3_ superlattice (see Supplementary Information) following ref. 4, and the results clearly demonstrate that LiTaO_3_ can polarize under *D* = 0, even down to extreme thin layers. These results further confirm that they are hyperFEs. We note that in a recent work^28^, Fu proposed some constrains in hyperFEs, i.e., 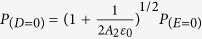 with *A*_2_*ε*_0_ < −0.75, using a fourth-order Ginzburg-Landau energy expansion in polarization *P*. However, both the Li*B*O_3_ compounds and the previously proposed *ABC* hexagonal hyperFEs^4^ do not satisfy these constrains, suggest that the fourth-order model in ref. 28 is over simplified, and higher order terms must be included in the free energy expansion.

## Discussion

We have shown that the large electronic dielectric constant and small effective charges may not be the necessary condition for the hyperFEs. This raises an interesting question: what are responsible for it? To answer this question, we start from a simplified effective Hamiltonian for FEs on an infinite lattice following ref. 23,


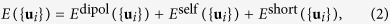


where **u**_*i*_ are the local normal modes at *i*-th cell. *E*^dipol^ represents the long-range dipole-dipole interaction, whereas *E*^self^, *E*^short^ are the energies of isolated local modes, and the short-range interactions between the local modes respectively. For the simplicity of discussion, we neglect the elastic energies, and their coupling to the local modes. Without losing generality, we further assume that the crystal has simple cubic structure.

First, let’s look at the dipole-dipole interactions,


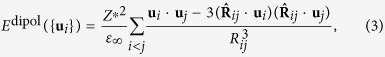


where *ε*_∞_ is the optical dielectric constant of the material. *R*_*ij*_ = |**R**_*ij*_| is the distance between the two local modes, where **R**_*ij*_ = **R**_*i*_ − **R**_*j*_ and 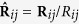. Direct evaluation of Eq. 3 in real space converges very slowly. Equation 3 can be evaluated using Ewald summation techniques. For simple cubic structure of infinite lattice size, the summation have been obtained in ref. 29. It turns out that *E*^dipol^ is non-analytic when **q** → 0,


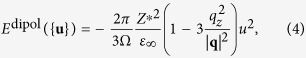


where *u* = |**u**| and Ω is the unit cell volume. Here, we assume that the phonon displacements are along the *z* axis. The short-range interactions can be obtained by setting Z* → 0, or *ε*_∞_ → ∞. The self-energy and the energy due to short-range interactions can thus be written in the following form as **q** → 0,


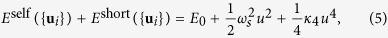


where 
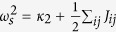
, *κ*_2_ is the on-site enenrgy contribution, and *J*_*ij*_ are the coupling constants between local modes **u**_*i*_ and **u**_*j*_. Therefore, the phonon frequency of the TO mode can be calculated as *q*_*x*_, *q*_*y*_ → 0,


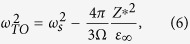


and the phonon frequencies of LO modes can be obtained as,


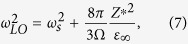


i.e., 
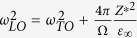
. Assuming that the eigenvectors of LO modes do not change much from that of TO modes, we can estimate 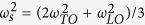. More generally, 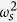 can be obtained by solving the following dynamic matrix if there are more than one phonon modes in a unit cell, which is applicable to any lattice symmetry^19^,


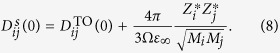


We calculate *ω*_*s*_ for typical perovskite ferroelctrics as well as Li*B*O_3_ using Eq. 8, where the Born effective charges Z*, and dielectric constants are same to those used in the LO phonon calculations, all obtained from first-principles calculations. The softest *ω*_*s*_ for these compounds are listed in Table 2. As we can see from the Table, in traditional perovskite FEs, such as PbTiO_3_, BaTiO_3_ etc., *ω*_*s*_ are all stable, meaning that the short-range interactions favorite the high symmetry non-polar structures. However, because of the large Born effective charges and small optical dielectric constants *ε*_∞_ in these materials, the long-range Coulomb interactions (the second term in the right hand of Eq. 6) overcome the short-range repulsive interactions 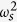, and the TO phonon mode frequencies become soft. In these materials, LO modes 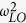 are all positive because 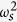 are already positive. These results are consistent with those of ref. 1, and the early point of view that the long-range Coulomb interactions are the driven forces for the FE states^23^.

However, for Li*B*O_3_, because the LO modes are soft (i.e., 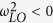), it easy to see from Eq. 7 that 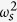 must also be negative. This suggests that the short-range interactions already favor the symmetry-broken polarized state in these materials. We therefore obtain one of the most important results of this paper: hyperFEs are a class of FEs, where the short-range interactions already favorite the symmetry broken polar states. This is a general feature of hyperFEs, or more precisely, a necessary condition for hyperFEs. This could happen in materials, e.g., Li*B*O_3_, where the ions have large size mismatches. Since the hyperferroelectricity comes from short-range local interactions, hyperFEs are not sensitive to the electric boundary conditions. Especially, LiOsO_3_ can be viewed as a special hyperFEs, in which *ε*_∞_ → ∞. More generally, any FE instability in a metal is a limiting case of hyperFE. It is interesting to see if more such metals can be found in searching for novel hyperFEs.

To conclude, we have shown that Li*B*O_3_ (*B* = V, Nb, Ta, Os) belong to the recently proposed hyperFEs, despite that some of them (LiNbO_3_ and LiTaO_3_) have large band gaps and Born effective charges. By resorting to an effective Hamiltonian model, we clarify that the origin of the hyperFEs is due to the structural instability driven by the short-range interactions. At least one route to find hyperFEs is to search in materials with large ion size mismatches. This work therefore provides a useful guidance in searching for novel hyperFEs.

## Methods

The electronic and structural properties of Li*B*O_3_ are calculated using density functional theory within local density approximation (LDA), implemented in the Vienna ab initio simulations package (VASP)^30^,^31^. The projector augmented-wave (PAW) pseudopotentials^32^ with a 500 eV plane-wave cutoff are used. The Brillouin zone is sampled with a 8 × 8 × 8 Monkhorst-Pack k-point grid converges the results very well. We relax the structure until the remaining forces are less than 1 meV/Å. Phonon frequencies are calculated using a finite difference method as implemented in Phonopy package^33^. The Born effective charges and the optical dielectric constants are calculated using density functional perturbation theory (DFPT)^34^. The above properties of Li*B*O_3_ are also calculated using other functionals, including GGA and LDA + U. Although the exact numbers of the results may vary, the main conclusions remain unchanged.

## Additional Information

**How to cite this article**: Li, P. *et al.* The origin of hyperferroelectricity in Li*B*O_3_ (*B* = V, Nb, Ta, Os). *Sci. Rep.* **6**, 34085; doi: 10.1038/srep34085 (2016).

## Supplementary Material

Supplementary Information

## Figures and Tables

**Figure 1:**
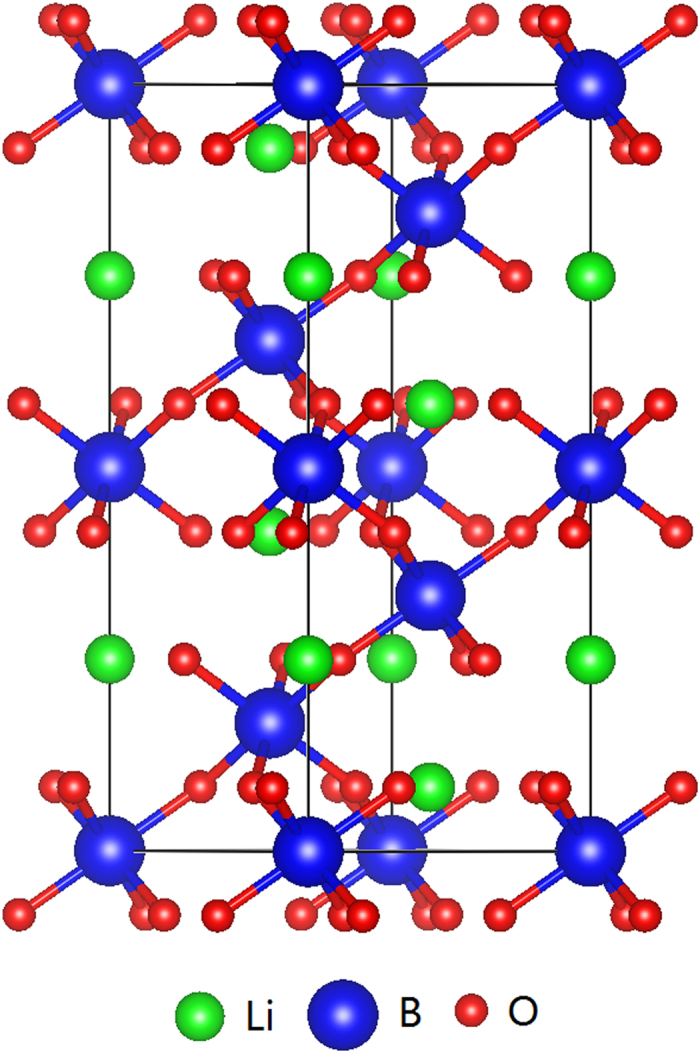
The hypothetical paraelectric (PE) structure of Li*B*O_3_ resulting from an average of the disordered structure above *T*_*c*_.

**Table 1:** Calculated band gaps, optical dielectric constants and atomic Born effective charges of Li*B*O_3_ in the PE and FE phases.

	gap (eV)	*ε*_∞_	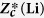	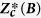	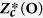
LiVO_3_ (PE)	0.4	18.8	1.13	13.36	−4.83
LiVO_3_ (FE)	1.9	6.4			
LiNbO_3_ (PE)	2.2	7.3	1.09	9.37	−3.49
LiNbO_3_ (FE)	2.9	5.8			
LiTaO_3_ (PE)	3.0	5.8	1.10	8.36	−3.15
LiTaO_3_ (FE)	3.6	5.3			
LiOsO_3_	0	∞			

**Table 2:** Calculated phonon frequencies of the softest TO modes (*ω*_*TO*_), LO modes (*ω*_*LO*_) and the phonon modes due to pure short-range interactions (*ω*_*s*_, calculated using Eq. 8).

	*ω*_*TO*_ (cm^−1^)	*ω*_*LO*_ (cm^−1^)	*ω*_*s*_ (cm^−1^)	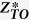	*P*_*E*_ = 0 (C/m^2^)	*P*_*D*_ = 0 (C/m^2^)
LiVO_3_	409*i*	160*i*	160*i*	20.1	1.79	0.29
LiNbO_3_	208*i*	104*i*	125*i*	8.9	1.00	0.08
LiTaO_3_	188*i*	77*i*	110*i*	5.8	0.72	0.05
LiOsO_3_	183*i*	183*i*	183*i*	—	—	—
PbTiO_3_	119*i*	105	95	7.5	0.57	0
BaTiO_3_	91*i*	181	180	10.1	0.12	0
NaNbO_3_	167*i*	81	73	8.7	0.49	0
KNbO_3_	143*i*	172	171	10.8	0.29	0

Also shown are the mode effective charges 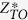 of the TO modes. and the electric polarization under 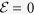 and *D* = 0. The values for PbTiO_3_, BaTiO_3_, NaNbO_3_, and KNbO_3_ are obtained under their cubic structures.
