# Supplementary material for: The origin of hyperferroelectricity in LiBO3 (B = V, Nb, Ta, Os)
Source: Sci Rep. 2016 Oct 3;6:34085. doi: 10.1038/srep34085 (PMC5046123; doi:10.1038/srep34085)
Supplement: Supplementary Information [file srep34085-s1.pdf]

Supplemental Material

Pengfei Li, Xinguo Ren, Guang-Can Guo, and Lixin He*

Key Laboratory of Quantum Information, University of Science and Technology of China, Hefei, 230026, China and Synergetic Innovation Center of Quantum Information and Quantum Physics, University of Science and Technology of China, Hefei, 230026, China

(Dated: April 18, 2016)

I. PHONON SPECTRA OF $LINBO_3$ AND $LITAO_3$

We show in Fig. S1(a) and Fig. S1(b), the phonon dispersions of high symmetry ($R\bar{3}c$) $LiNbO_3$ and $LiTaO_3$ for **q** along X-Γ-Z directions. The phonon frequencies are calculated using a finite difference method as implemented in Phonopy package[1] interfaced with VASP.[2,3] To calculate the phonon dispersion in the full Brillouin Zone, we use a 2×2×2 supercell, containing 80 atoms. The TO modes are obtained for **q** along the X-Γ, whereas LO modes are obtained for **q** along the Γ-Z direction. The frequencies of A_{2u} LO modes at the Γ point obtained using the supercell method[1] are in excellent agreement (within few wave numbers) with those calculated from Eq. (1) in the main text.

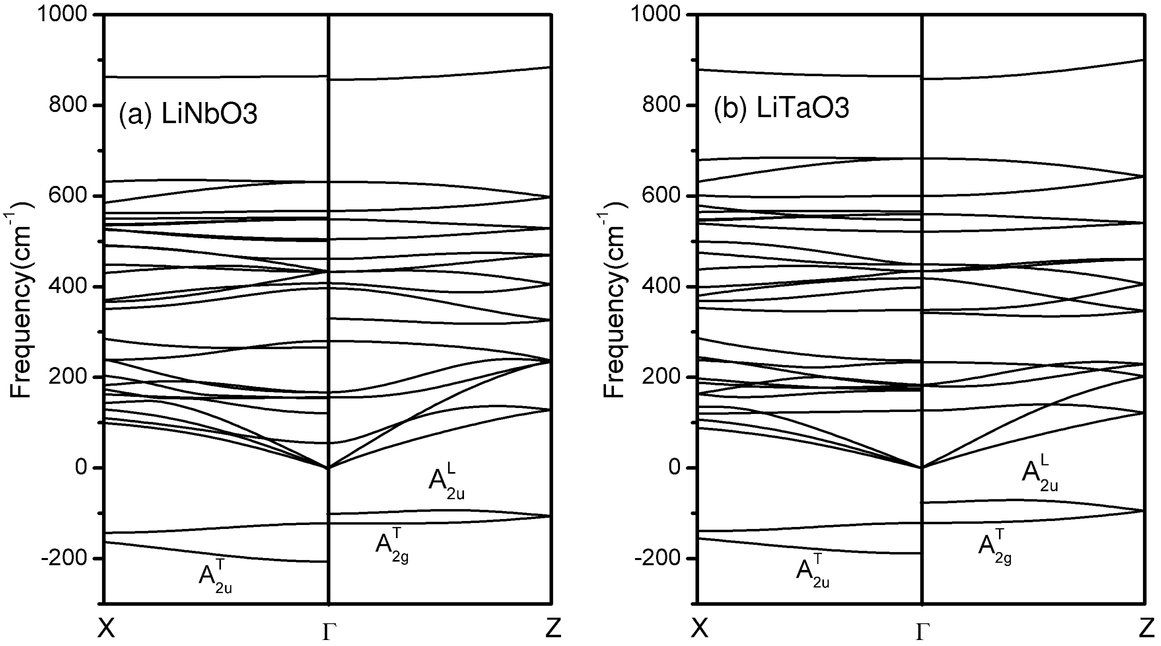

FIG. S1: Phonon dispersions along X-Γ-Z directions for high symmetry ($R\bar{3}c$) (a) $LiNbO_3$ and (b) $LiTaO_3$.

II. $LITAO_3$ SUPERLATTICE

To further check if $LiBO_3$ can polarize under the D=0 boundary condition,[4] we perform first-principles calculations on an artificial $LiTaO_3$ superlattice as schematically shown in Fig. S2 (a). The superlattice is constructed by 5 layers of high symmetry ($R\bar{3}c$) $LiTaO_3$ unit cells with periodic boundary condition. Each unit cell contains 30 atoms as

depicted in Fig. 1 of the main text. We allow the atoms of one layer in the superlattice plus the interfacial atoms of this layer [i.e., the region between the red dashed lines in Fig. S2(a)] to relax freely while keep all atoms in the rest 4 layers fixed at their high symmetric positions. The atoms in the fixed layers are at their high symmetry positions and therefore these layers are non-polar, i.e., P=0, which enforce the $D \approx 0$ boundary condition.[4] Indeed one finds an unstable polar mode when relaxing the superlattice structure. We plot the energy verses the atomic displacement of polar mode, and the results are shown in Fig. S2 (b), which clearly show a double well structure. This polar mode pattern is very similar to that the of bulk LO mode for $LiTaO_3$. We also calculate the electric polarization for the structure at energy minimum, and the obtained electric polarization is P=0.07 C/m^2. The results clearly demonstrate that $LiTaO_3$ can polarize under D=0, even down to extreme thin layers.

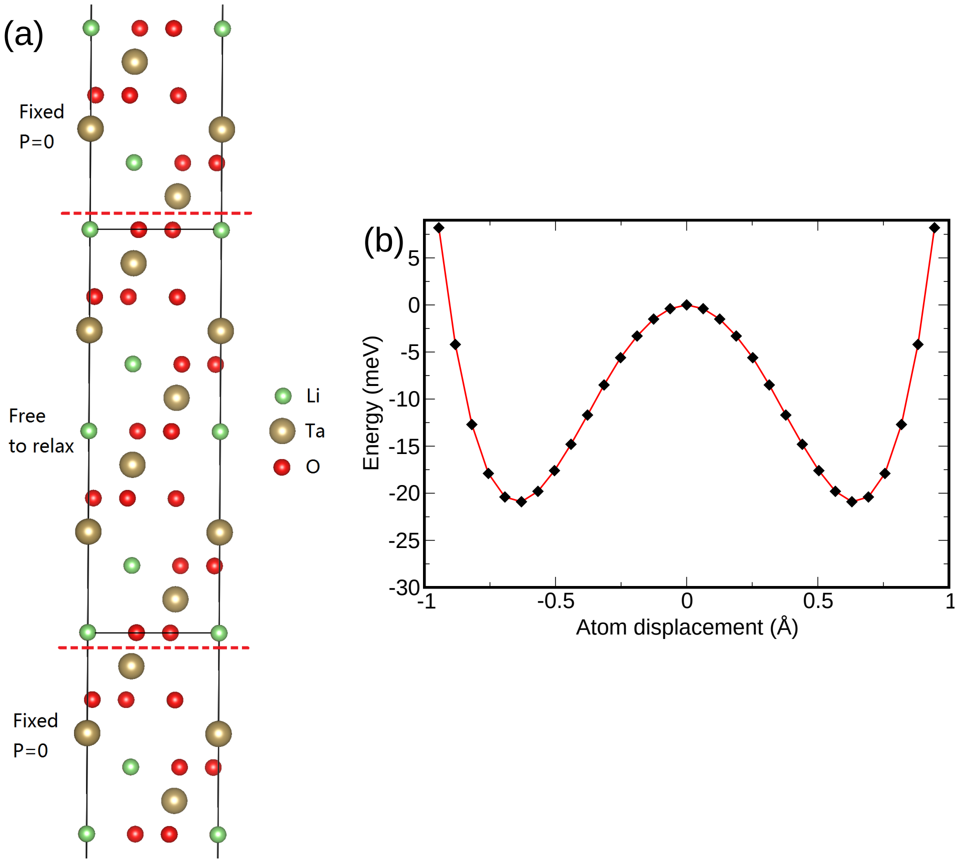

FIG. S2: (a) Schematic diagram of the artificial $LiTaO_3$ superlattice. The superlattice is constructed by 5 layers of high-symmetry ($R\bar{3}c$) $LiTaO_3$ unit cells with periodic boundary condition. We allow the atoms in the middle layer plus the interfacial atoms, i.e., the region between the red dashed lines, to relax freely while keeping all other atoms fixed at their high symmetric positions. (b) The total energy of the superlattice as a function of the polar phonon displacement.

* Electronic address: helx@ustc.edu.cn.

[1] A. Togo, F. Oba, and I. Tanaka, Phys. Rev. B **78**, 134106 (2008).
[2] G. Kresse and J. Hafner, Phys. Rev. B **47**, R558 (1993).
[3] G. Kresse and J. Furthmuller, Phys. Rev. B **54**, 11169 (1996).
[4] K. F. Garrity, K. M. Rabe, and D. Vanderbilt, Phys. Rev. Lett. **112**, 127601 (2014).
